# The Interactions of Magnesium Sulfate and Cromoglycate in a Rat Model of Orofacial Pain; The Role of Magnesium on Mast Cell Degranulation in Neuroinflammation

**DOI:** 10.3390/ijms24076241

**Published:** 2023-03-26

**Authors:** Dragana Srebro, Branko Dožić, Sonja Vučković, Katarina Savić Vujović, Branislava Medić Brkić, Ivan Dožić, Milorad Srebro

**Affiliations:** 1 Department of Pharmacology, Clinical Pharmacology and Toxicology, Faculty of Medicine, University of Belgrade, Dr Subotića-Starijeg 1, 11129 Belgrade, Serbia; srebrodragana1@gmail.com (D.S.);; 2 Department of Pathology, School of Dental Medicine, University of Belgrade, Dr Subotića-Starijeg 1, 11000 Belgrade, Serbia; 3 Department of Biochemistry, School of Dental Medicine, University of Belgrade, Dr Subotića-Starijeg 1, 11000 Belgrade, Serbia

**Keywords:** trigeminal pain, mast cell, neuroinflammation, nociception, magnesium, cromoglycate, preemptive analgesia, drug interactions

## Abstract

Mast cell degranulation impacts the development of pain and inflammation during tissue injury. We investigated the antinociceptive effect of a combination of cromoglycate and magnesium in the orofacial model of pain and the histological profile of the effect of magnesium in orofacial pain. In male Wistar rats, formalin (1.5%, 100 µL) was injected subcutaneously into the right upper lip of rats after cromoglycate and/or magnesium. Pain was measured as the total time spent on pain-related behavior. Toluidine blue staining was used to visualize mast cells under the light microscope. In the formalin test, in phase 1, magnesium antagonized the antinociceptive effect of cromoglycate, while in phase 2, it potentiated or inhibited its effect. Magnesium significantly reduced mast cell degranulation in the acute phase by about 23% and in the second phase by about 40%. Pearson’s coefficient did not show a significant correlation between mast cell degranulation and pain under treatment with magnesium. The cromoglycate–magnesium sulfate combination may prevent the development of inflammatory orofacial pain. The effect of a combination of cromoglycate–magnesium sulfate depends on the nature of the pain and the individual effects of the drugs. Magnesium reduced orofacial inflammation in the periphery, and this effect did not significantly contribute to its analgesic effect.

## 1. Introduction

Orofacial pain refers to any pain felt in the mouth, jaw, and/or face. Facial pain is defined as pain in the face that occurs below the orbital and above the neckline [1]. Facial pain syndrome can be a consequence of certain dental interventions [2]. Trigeminal neuropathy includes non-dental forms of orofacial pain, such as trigeminal neuralgia, orofacial migraine, temporomandibular disorders, burning mouth syndrome, and salivary gland pain [3,4]. These forms of trigeminal neuralgia are considered variations of chronic orofacial pain [5]. Due to its chronic nature, the prevention of the development of orofacial trigeminal pain is especially important. The main problems in the treatment of orofacial pain are the diversity of the nature of pain, resistance to pharmacotherapy, and the frequent side effects of pharmacotherapy. In recent years, research has focused on the orofacial pain pathway and new therapeutic options for the prevention of the development of orofacial pain.

Mast cells are mobile immune cells that contain granules with mediators. The cells reside in connective, mucosal/epithelial tissue near blood vessels and sensory nerve endings. They can be activated by immunological (IgE) and nonimmunological (e.g., nerve injury) mechanisms [6]. After degranulation, mast cells release mediators (e.g., histamine, serotonin, interleukins, tumor necrosis factor alpha), which can produce inflammation and sensitization of the sensory neurons [7,8,9]. The cutaneous neurogenic inflammation caused by the degranulation of mast cells is augmented through the action of sensory skin nerves that release neuropeptides and further increase mast cell degranulation [10]. This can create positive feedback that supports the transition from acute to chronic, and to neuropathic pain [11]. Therapies that target mast cell functions may be essential for many painful and inflammatory disorders in the orofacial region [12,13]. Besides immune-mediated mechanisms in pain, mast cells also can mediate in pruriceptive activation [14]. Mast cells can thus have a significant molecular effect on neuroinflammation, which consequently leads to the development of peripheral and then central sensitization [15].

The orofacial formalin test serves as a translational model for spontaneous pain in the orofacial region and with trigeminal pain associated with tissue injury and inflammation [16]. Phase 1 is the result of the direct activation of peripheral nociceptors, while phase 2 is the result of inflammation and central sensitization [17,18]. In the formalin hind-paw test, formalin can directly cause mast cell degranulation [19], leading to pain associated with the simultaneous endogenous release of histamine and serotonin [8]. Up to now, mast cell degranulation has not been studied in the orofacial formalin model of pain. In addition, the effect of magnesium on cell degranulation has not been studied.

Sodium cromoglycate or cromolyn is a drug that belongs to the group of mast cell membrane stabilizers. Its main use is as a prophylactic drug in asthma treatment. Recent data have shown that either alone or in combination with nonsteroidal anti-inflammatory drugs, cromoglycate exhibits anti-diabetic and anti-cancer properties [20,21]. In addition, cromoglycate displayed an analgesic effect in different models of neuropathic and inflammatory pain [8,13,22].

Our previous results showed that when magnesium is applied as the sole drug, it exerts an analgesic effect in different models of pain during inflammation, as well as in orofacial pain [16,23,24,25,26]. The effect occurs after systemic, not after local administration [23]. In combination with opioids or N-methyl-D-aspartate (NMDA) antagonists, magnesium increases their analgesic effect in inflammatory and neuropathic pain [26,27,28]. The mechanisms of the analgesic effect of magnesium include the blockade of NMDA receptors, modulation of the production of nitric oxide, and activation of transient receptor potential (TRP) channels vanilloid type 1 and 4 and ankyrin type 1 [24,25,26,29,30]. Recent data show that peripheral NMDA receptors located on peripheral sensory trigeminal ganglion neurons have magnesium-dependent mechanisms of action, contributing to the pathophysiology of migraines [31].

In this study, we hypothesized that magnesium, as a known calcium channel blocker, can stabilize the mast cell membrane and reduce mast cell degranulation, which could be one of the mechanisms of its effect on neuroinflammation and its analgesic effect. In this way, magnesium could interact with cromoglycate and potentiate its analgesic effect. To test these hypotheses, the present study primarily aimed to identify the effect of magnesium on mast cell degranulation as one possible mechanism of the anti-inflammatory and analgesic effect of magnesium in the orofacial formalin test in rats. Secondly, we investigated the interaction between cromoglycate and magnesium sulfate as a potential benefit of therapeutic combination.

## 2. Results

### 2.1. The Effects of Cromoglycate and Magnesium Sulfate Alone in the Formalin Orofacial Test in Rats

**Figure 1:**
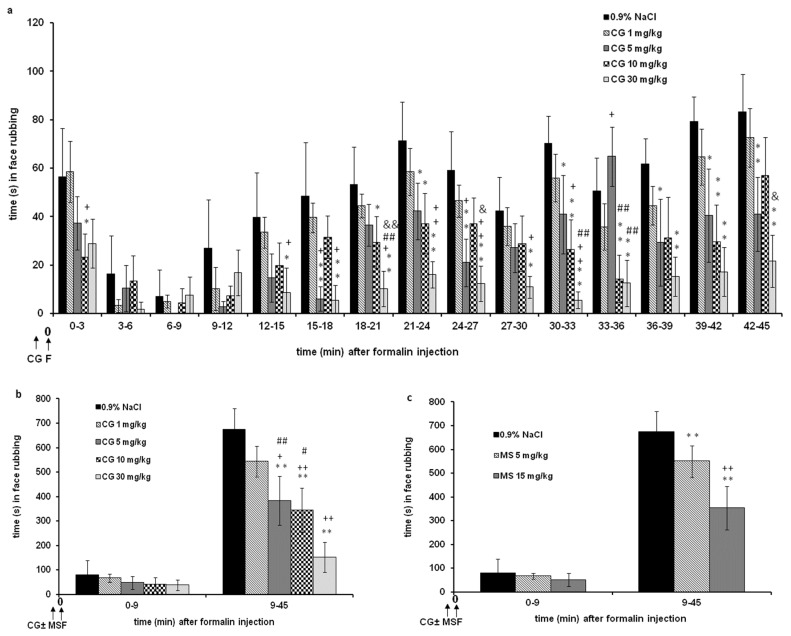
The antinociceptive effect of cromoglycate (CG) (**a**,**b**) or magnesium sulfate (MS) (**c**) in the formalin orofacial test in rats. Each bar represents the mean time (in seconds) of face rubbing ± SD observed in 6 rats. Time intervals of nociceptive response (face rubbing) in 3 min intervals are shown in graph (**a**), and the total time spent in the nociceptive response in phase 1 (0–9 min) and phase 2 (9–45 min) in the formalin test is shown in graph (**b**) or (**c**). Statistical significance (two-way ANOVA followed by Tukey’s HSD) was determined by comparing with the control (0.9% NaCl; *n* = 6) (* *p* < 0.05, ** *p* < 0.01), ^+^ *p* < 0.05, ^++^ *p* < 0.05 in comparison with CG 1 (*n* = 6), ^#^ *p* < 0.05, ^##^ *p <* 0.01 in comparison to CG 5 (*n* = 6) (**a**) or 30 (*n* = 6) (**b**), ^&^ *p* < 0.05, ^&&^ *p* < 0.01 in comparison to CG 10 (*n* = 6). Statistical significance (two-way ANOVA followed by Tukey’s HSD) was determined by comparing with the control (0.9% NaCl; *n* = 6) (** *p* < 0.01) or between MS (*n* = 6 per group, total 12) (^++^ *p* < 0.05 in comparison with MS 5).

Systemic cromoglycate (1–30 mg/kg; s.c.) produced a statistically significant and dose-dependent decrease in the total time spent exhibiting pain-related behavior (F = 3.385; *p* < 0.001; for intervals). There is a significant interaction (F = 29.329; *p* < 0.001; for intervals) between the observed time and the different doses of cromoglycate (Figure 1a). There are significant differences between the doses during the observed time in most intervals (F = 19.931; *p* < 0.001). Statistical significance was observed between cromoglycate 1, 5, and 10 mg/kg and cromoglycate 30 mg/kg and between 1 and 30 mg/kg. Cumulatively, the antinociceptive effect of cromoglycate is significant only in phase 2 (F = 33.392; *p* < 0.001; for phases). Cumulatively, the maximum antinociceptive effect of cromoglycate was 52.4 ± 10.4% during phase 1 and 77.4 ± 3.7% during phase 2 and was achieved at a dose of 30 mg/kg (Figure 1b).

Systemic magnesium sulfate at doses of 5 and 15 mg/kg (s.c.) significantly reduced pain behavior only in phase 2 (F = 327.452; *p* < 0.001). Similar maximal effect was achieved with 5 and 15 mg/kg, 41.8 ± 4.0% and 40.0 ± 4.2%, respectively. In phase 1, magnesium sulfate at doses of 5 and 15 mg/kg reduced nociception by about 20–50%. The effect was not statistically significant (*p* = 0.688 and *p* = 0.543, respectively) (Figure 1c). In comparison to the formalin-treated group, in the group of non-treated (naive) rats, face rubbing was sporadic (about 8 s during 10 min of observation and about 10 s during 10–45 min of observation).

### 2.2. The Effect of the Combination of Cromoglycate and Magnesium Sulfate in the Formalin Orofacial Test in Rats

#### 2.2.1. Different Doses of Cromoglycate (1, 5, and 10 mg/kg) Were Combined and Tested with Low-Effective Dose of Magnesium Sulfate (5 mg/kg) (Figure 2)

**Figure 2:**
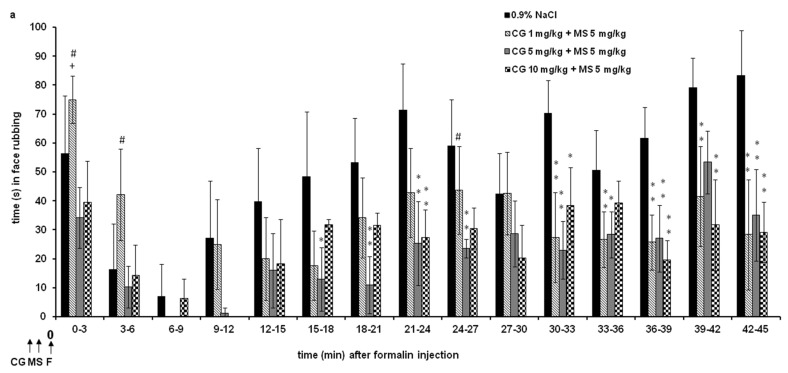

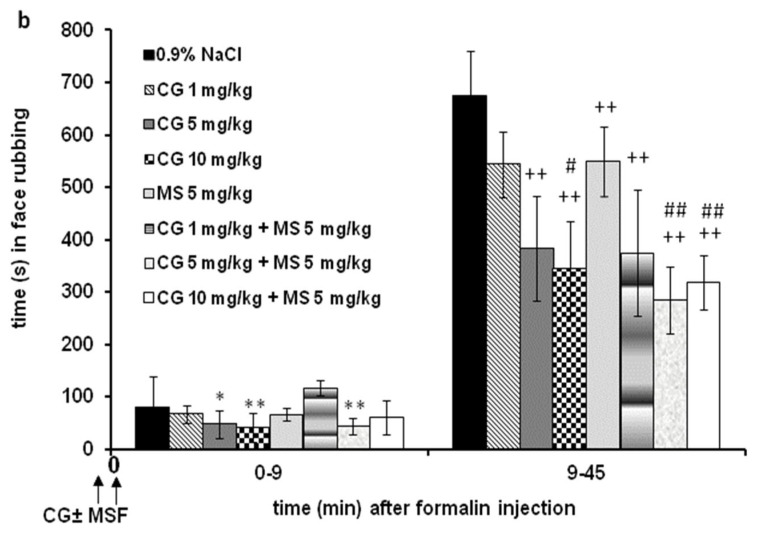
The antinociceptive effect of cromoglycate (CG)–magnesium sulfate (MS 5 mg/kg) combination in the formalin orofacial test in rats. Each bar represents the mean time (in seconds) of face rubbing ± SD observed in 6 rats. Time intervals of nociceptive response (face rubbing) in 3 min intervals are shown in graph (**a**), and the total time spent in the nociceptive response in phase 1 (0–9 min) and phase 2 (9–45 min) in the formalin test is shown in graph (**b**). (**a**) Statistical significance (two-way ANOVA followed by Tukey’s HSD) was determined by comparing with the control (0.9% NaCl; *n* = 6) (* *p* < 0.05, ** *p* < 0.01), ^+^ *p* < 0.05, ^++^ *p* < 0.01 in comparison with CG 5 (*n* = 6) or 10 (*n* = 6), ^#^ *p* < 0.05 in comparison with CG 5 + MS 5 (*n* = 6). (**b**) Statistical significance (two-way ANOVA followed by Tukey’s HSD) was determined by comparing with the control (0.9% NaCl; *n* = 6) (^++^ *p* < 0.01), * *p* < 0.05, ** *p* < 0.01 in comparison with CG 1 + MS 5 (*n* = 6), ^#^ *p* < 0.05, ^##^ *p* < 0.01 in comparison with CG 1 (*n* = 6).

All combinations significantly changed total nociception (F = 2.686; *p* < 0.001), and there is a significant interaction (F = 15.267; *p* < 0.001) during observed times and the changes in the effects of the combination of drugs (Figure 2a). The effect of the cromoglycate (1 mg/kg)–magnesium sulfate (5 mg/kg) combination was significantly lower compared to the cromoglycate (10 mg/kg)–magnesium sulfate (5 mg/kg) combination at time points of 0–3 (*p* = 0.033) and 3–6 (*p* = 0.031) min. All combinations produced a significant antinociceptive effect compared to the control (0.9% NaCl) in most 3 min intervals from 18 to 45 min (F = 466.880; *p* < 0.001). Cumulatively, all combinations achieved a significant antinociceptive effect only in phase 2 (F = 11.462; *p* < 0.001) (Figure 2b). In phase 1, the combinations did not produce a significant effect when compared to the controls, but there was a significant difference between the combinations themselves. In phase 1, the effect of the cromoglycate 5 mg/kg–magnesium sulfate 5 mg/kg combination was significantly higher compared to cromoglycate 1 mg/kg–magnesium sulfate 5 mg/kg (*p* = 0.009) (Figure 2b).

#### 2.2.2. Different Doses of Cromoglycate (1, 10, and 30 mg/kg) Were Combined and Tested with High Effective Dose of Magnesium Sulfate (15 mg/kg) (Figure 3)

**Figure 3:**
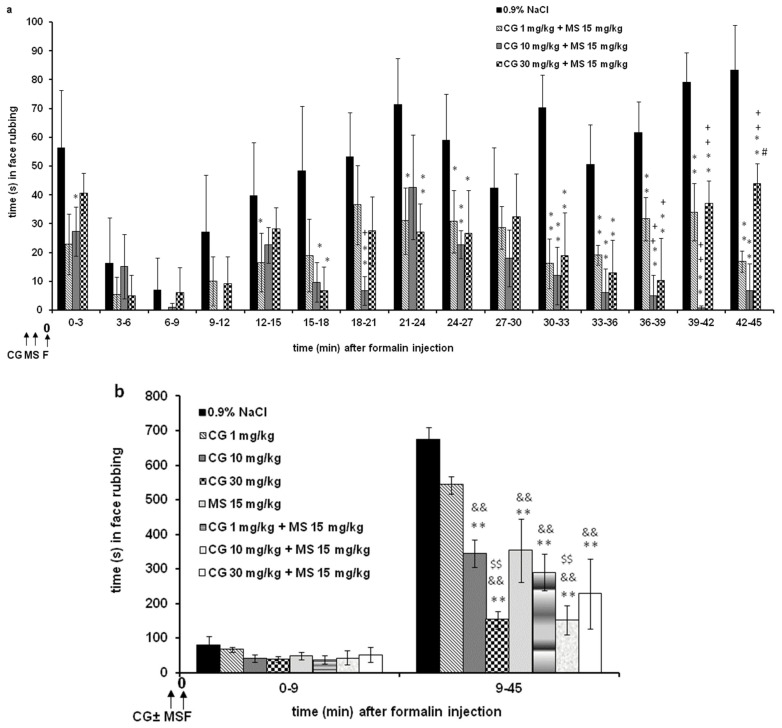
The antinociceptive effect of cromoglycate (CG)–magnesium sulfate (MS 15 mg/kg) in the formalin orofacial test in rats. Each bar represents the mean time (in seconds) of face rubbing ± SD observed in 6 rats. Time intervals of nociceptive response (face rubbing) in 3 min intervals are shown in graph (**a**), and the total time spent in the nociceptive response in phase 1 (0–9 min) and phase 2 (9–45 min) in the formalin test is shown in graph (**b**). Statistical significance (two-way ANOVA followed by Tukey’s HSD) was determined by comparing with the control (0.9% NaCl; *n* = 6) (* *p* < 0.05, ** *p* < 0.01), ^+^ *p* < 0.05, ^++^ *p* < 0.01 in comparison with CG 1 + MS 15 (*n* = 6), ^#^ *p* < 0.05 in comparison with CG 10 + MS 15 (*n* = 6), ^&&^ *p* < 0.01 in comparison with CG 1 (*n* = 6), and ^$$^ *p* < 0.01 in comparison with CG 10 (*n* = 6).

All combinations significantly reduced nociception (F = 3.592; *p* < 0.001), and there is a significant interaction (F = 14.020; *p* < 0.001) because the effects of the combination of drugs are changed over 45 min (Figure 3a). All combinations produced a significant antinociceptive effect compared to the control (0.9% NaCl) in most 3 min intervals from 0 to 3 and 12 to 45 min (F = 34.253; *p* < 0.001). The combination of cromoglycate (30 mg/kg)–magnesium sulfate (15 mg/kg) produced a significantly (*p* < 0.05) lower effect compared to cromoglycate (1 and 10 mg/kg)–magnesium sulfate (15 mg/kg) combination at time points 18–21 and 36 to 42 min. Cumulatively, in phase 1, there were no significant differences between groups (Figure 3b). Cumulatively, all combinations achieved a significantly antinociceptive effect only in phase 2 (F = 600.156; *p* < 0.001) (Figure 3b). In phase 2, the effects of combinations of 1 (*p* < 0.001) and 10 (*p* = 0.009) mg/kg cromoglycate and magnesium sulfate 15 mg/kg were significantly higher when compared to the effects of 1 or 10 mg/kg cromoglycate alone (Figure 3b).

### 2.3. Interactions between Cromoglycate and Magnesium Sulfate

#### 2.3.1. Interaction between Cromoglycate and Magnesium Sulfate in Phase 1 of Formalin Test—Acute Nociceptive Orofacial Pain

In phase 1 of the test, the combination of a fixed-quantity dose of magnesium sulfate (5 or 15 mg/kg) along and 3 different doses (1, 5 and 10 mg/kg) of cromoglycate (fixed fractions of the median effective dose (ED50): 1/2ED50, 2ED50, 4ED50) were examined. In phase 1, the antinociceptive effect of cromoglycate showed dose-dependent, and the maximal effect of doses 1, 5, and 10 mg/kg was 33.0%, 66.6%, and 75%, respectively. The estimated ED50 value was 2.35 mg/kg.

**Figure 4:**
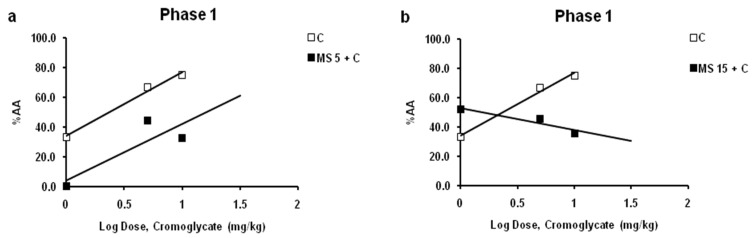
Interaction between cromoglycate (CG) and magnesium sulfate (MS) in phase 1 of formalin test. Log dose–response for CG (1, 5, 10 and 30 mg/kg; s.c.) alone and in the combination with MS (5 mg/kg) (**a**) or MS (15 mg/kg) (**b**) in phase 1. Data are expressed as a percent of antinociception (AA (%)).

Magnesium sulfate at a dose of 5 mg/kg inhibited the antinociceptive effects of cromoglycate (1–10 mg/kg) (Figure 4a). The inhibitory effects for cromoglycate doses 1, 5, and 10 mg/kg were 100%, 33.6%, and 56.5%, respectively. The log dose–response curves for cromoglycate administered alone, and cromoglycate administered with a fixed dose of magnesium sulfate were constructed and compared (Figure 4a). There was a rightward shift of the log dose–response regression curve for cromoglycate in the presence of magnesium sulfate, compared with the log dose–response regression curve for cromoglycate alone, which indicates antagonism. However, the shift is not statistically significant (*p* > 0.05; relative potency test) due to wide CL. The potency ratio was 2.40 (CL 0.50–139.12). The slopes are not significantly different (*p* < 0.05; test for parallelism). The ED50 of cromoglycate in combination was 5.64 mg/kg.

Magnesium sulfate at a dose of 15 mg/kg increased the subeffective (1 mg/kg) and decreased (10 and 30 mg/kg) the antinociceptive effects of cromoglycate (Figure 4b). For a cromoglycate dose of 1 mg/kg, the increasing effect was about 20%. The inhibitory effects for cromoglycate doses 10 and 30 mg/kg were 31.8% and 52.4%, respectively. The log dose–response curves for cromoglycate administered alone and cromoglycate administered with a fixed dose of magnesium sulfate were constructed and compared (Figure 4b). Although there was a rightward shift of the log dose–response regression curves, the slopes between lines are different, lines are not parallel, and interaction is not possible to determine (*p* > 0.05; test for parallelism).

#### 2.3.2. Interaction between Cromoglycate and Magnesium Sulfate in Phase 2 of Formalin Test—Acute Inflammatory Orofacial Pain

In the phase 2 of test the combination of a fixed quantity dose of magnesium sulfate (5 or 15 mg/kg) along and 3 different doses (1, 10 and 30 mg/kg) of cromoglycate (fixed fractions of the ED50: 1/10ED50, 1ED50, 4ED50) were examined. In phase 2 the antinociceptive effect of cromoglycate show dose-dependent effect and maximal effect up to 100%. The respective ED50 value was 7.00 mg/kg.

**Figure 5:**
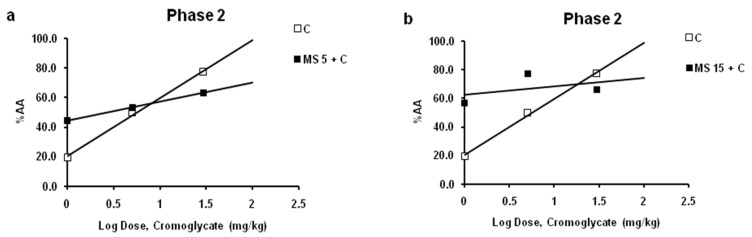
Interaction between cromoglycate (CG) and magnesium sulfate (MS) in phase 2 of formalin test. Log dose–response for CG (1, 10, and 30 mg/kg; sc) alone and in the combination with MS (5 mg/kg) (**a**) or MS (15 mg/kg) (**b**) in phase 2. Data are expressed as a percent of antinociception (AA (%)).

Magnesium sulfate at dose of 5 mg/kg increased the antinociceptive effect of the low effective (1 mg/kg), and decreased the effect of the high effective (10 mg/kg) dose of cromoglycate (Figure 5a). For a cromoglycate dose of 1 mg/kg, the increasing effect was about 25%. The inhibitory effect for cromoglycate doses 30 mg/kg was 18.3%. The log dose–response curves for cromoglycate administered alone and cromoglycate administered with a fixed dose of magnesium sulfate were constructed and compared (Figure 5a). Although there was a leftward shift of the log dose–response regression curve for cromoglycate in the presence of magnesium sulfate slopes are different, lines are not parallel, and interaction cannot be determined (*p* > 0.05; test for parallelism).

Magnesium sulfate at a dose of 15 mg/kg increased the antinociceptive effect of the low and medium effective (1 and 10 mg/kg) doses and decreased the effect of the high effective (30 mg/kg) dose of cromoglycate (Figure 5b). For cromoglycate doses of 1 and 10 mg/kg, the increasing effect was about 37% and 28%, respectively. The inhibitory effect for cromoglycate doses of 30 mg/kg was 14.6%. The log dose–response curves for cromoglycate administered alone and cromoglycate administered with a fixed dose of magnesium sulfate were constructed and compared (Figure 5b). Although there was a leftward shift of the log dose–response regression curve for cromoglycate in the presence of magnesium sulfate, slopes are different, lines are not parallel, and interaction is not possible to determine (*p* > 0.05; test for parallelism).

### 2.4. Magnesium Sulfate Reduced the Total Number of Mast Cells in Orofacial Region in the Formalin-Induced Orofacial Pain Test

**Figure 6:**
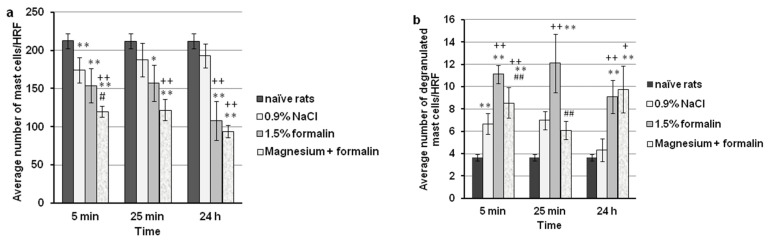
The effect of magnesium sulfate (MS) on the total (**a**) and degranulated (**b**) number of mast cells in the upper lip dermis in the formalin orofacial test in rats. Each bar represents the average number of total or degranulated mast cells per high representative field (HRF) ± SD obtained from 6 rats. Mast cells counted pathohistologically by toluidine blue staining at high magnification (400×), before (naive rats; *n* = 6) and after (5 min, 25 min, and 24 h) injection of formalin (6 rats per 3 time points; *n* = 18) with/without magnesium (6 rats per 3 time points; *n* = 18) or 0.9% NaCl (6 rats per 3 time points; *n* = 18). (**a**) Statistical significance (two-way ANOVA followed by Tukey’s HSD) was determined by comparing with naive rats (* *p* < 0.05, ** *p* < 0.01), ^++^ *p* < 0.01 in comparison with 0.9% NaCl, ^#^ *p* < 0.05 in comparison with formalin. (**b**) Statistical significance (two-way ANOVA followed by Tukey’s HSD) was determined by comparing with naive rats (** *p* < 0.01), ^+^ *p* < 0.05, ^++^ *p* < 0.01, respectively, in comparison with 0.9% NaCl, ^##^ *p* < 0.01 in comparison with formalin.

**Figure 7:**
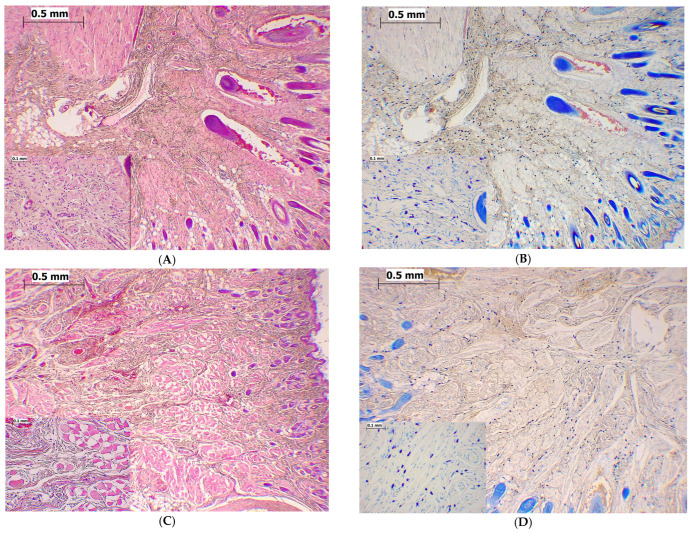

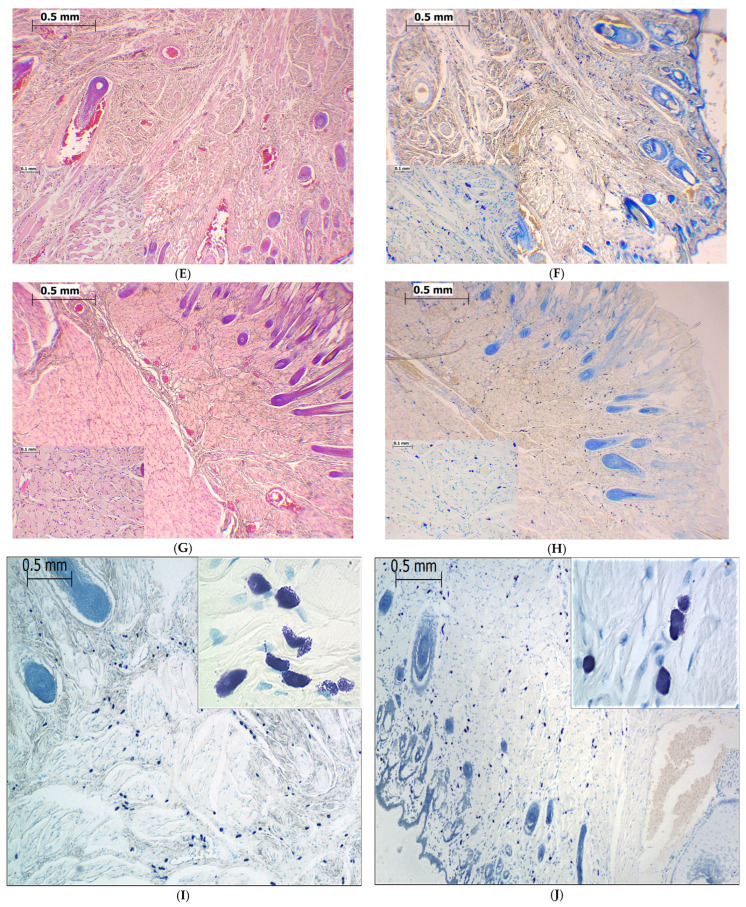
The effect of magnesium sulfate on morphology in the upper lip dermis in the formalin orofacial test in rats. Representative image of rat vibrissa pad histology stained with hematoxylin-eosin (HE) or toluidine blue in naive rats (*n* = 6 (**A**,**B**) images) or 25 min after vehicle (0.9%NaCl; *n* = 6; (**C**,**D**) images), formalin (*n* = 6; (**E**,**F**) images) and formalin + magnesium sulfate (*n* = 6; (**G**,**H**) images) injection. Magnesium sulfate (15 mg/kg, s.c.) injected systemically 5 min before formalin; formalin (1.5%, 0.1 mL) injected s.c. to the right upper lip. Depending on group at power ×40 (big images) or ×200 magnification (small images), there are thick (**A**–**D** images) or moderate (**E**–**H** images) mast cell infiltrates in rat vibrissae pad tissues. Microphotographs show positively stained mast cells with a blue-violet color. Degranulated mast cells in rat vibrissae pad tissues (×600) at 25 min are show in the formalin group ((**I**) image, small image) and the formalin + magnesium group ((**J**) image, small image). The length of the scale bar for big microphotographs and magnification at ×40 is 0.5 mm, and for small microphotographs at magnification ×200 is 0.1 mm. Images: (**A**)—Naive group, HE staining (40×, 200×); (**B**)—Naive group, toluidine blue staining (40×, 200×); (**C**)—0.9% NaCl group, HE staining (40×, 200×); (**D**)—0.9% NaCl group, toluidine blue staining (40×, 200×); (**E**)—Formalin group, HE staining (40×, 200×); (**F**)—Formalin group, toluidine blue staining (40×, 200×); (**G**)—-Formalin + magnesium group, HE staining (40×, 200×); (**H**)—Formalin + magnesium group, toluidine blue staining (40×, 200×); (**I**)—Degranulated mast cells (small image), formalin group, toluidine blue staining (100×, 600×); (**J**)—Degranulated mast cells (small image), formalin + magnesium group, toluidine blue staining (40×, 600×).

Histological analysis of the non-injected upper lip of rats revealed the presence of 212 ± 8 mast cells/high representative fields (HRF). As presented in Figure 6a, all treatments significantly decreased the total number of mast cells (F = 44.95; *p* < 0.001) in the upper lip and changed the pattern of this effect with time (F = 4.738; *p* = 0.015). Compared to the non-injected (naive) rats, the injection of formalin (1.5%, 100 μL, s.c.) in the upper lip caused a significant reduction in the total number of mast cells at the 5 min, 25 min, and 24 h time points (*p* < 0.001; *p* < 0.001; *p* < 0.001, respectively). A significant difference was observed between 0.9% injected NaCl and the non-injected upper lip only at the 5 min time point (*p* = 0.005). Compared to 0.9% NaCl, the injection of formalin (1.5%, 100 μL, s.c.) caused a significant (*p* < 0.001) decrease in the total number of mast cells in the upper lip at the 24 h time point by 43.9 ± 5.3%. Magnesium sulfate (15 mg/kg, s.c.) significantly potentiated the effect of formalin (*p* = 0.013) and of 0.9% NaCl (*p* < 0.001) at the 5 min time point. With HE staining, no or slight inflammatory infiltrate was found in rat vibrissae pad tissues treated with formalin and magnesium in phase 2 of the test. Representative micrographs of mast cell density in different groups are given in Figure 7.

### 2.5. Magnesium Sulfate Reduced Degranulation of Mast Cells in the Formalin-Induced Orofacial Pain Test

The orofacial dermis of naive rats had a smaller number of mast cells exhibiting spontaneous degranulation (mean number 3/HRF). As presented in Figure 6b, all treatments significantly changed the degranulation of mast cells in the upper lip (F = 2360.58; *p* < 0.001) during the study period. Formalin (1.5%, 100 μL, s.c.) injected into the upper lip caused a statistically significant increase in mast cell degranulation at the 5 min, 25 min, and 24 h time points when compared to the non-injected upper lip (*p* < 0.001; *p* < 0.001; *p* = 0.001, respectively) or the 0.9% NaCl-injected upper lip (*p* < 0.001; *p* = 0.007; *p* = 0.017, respectively). A significant difference was observed between 0.9% injected NaCl and non-injected upper lips only at the 5 min time point (*p* = 0.001). Magnesium sulfate at a dose of 15 mg/kg administered s.c. 5 min before the formalin-induced orofacial pain significantly reduced the number of degranulated mast cells by 23.0 ± 5.0% (*p* = 0.001) and 39.9 ± 11.0% (*p* = 0.008) at the 5 min and 25 min time points, respectively. Representative micrographs of degranulated mast cells are given in Figure 7I,J.

### 2.6. Correlation Analysis

**Figure 8:**
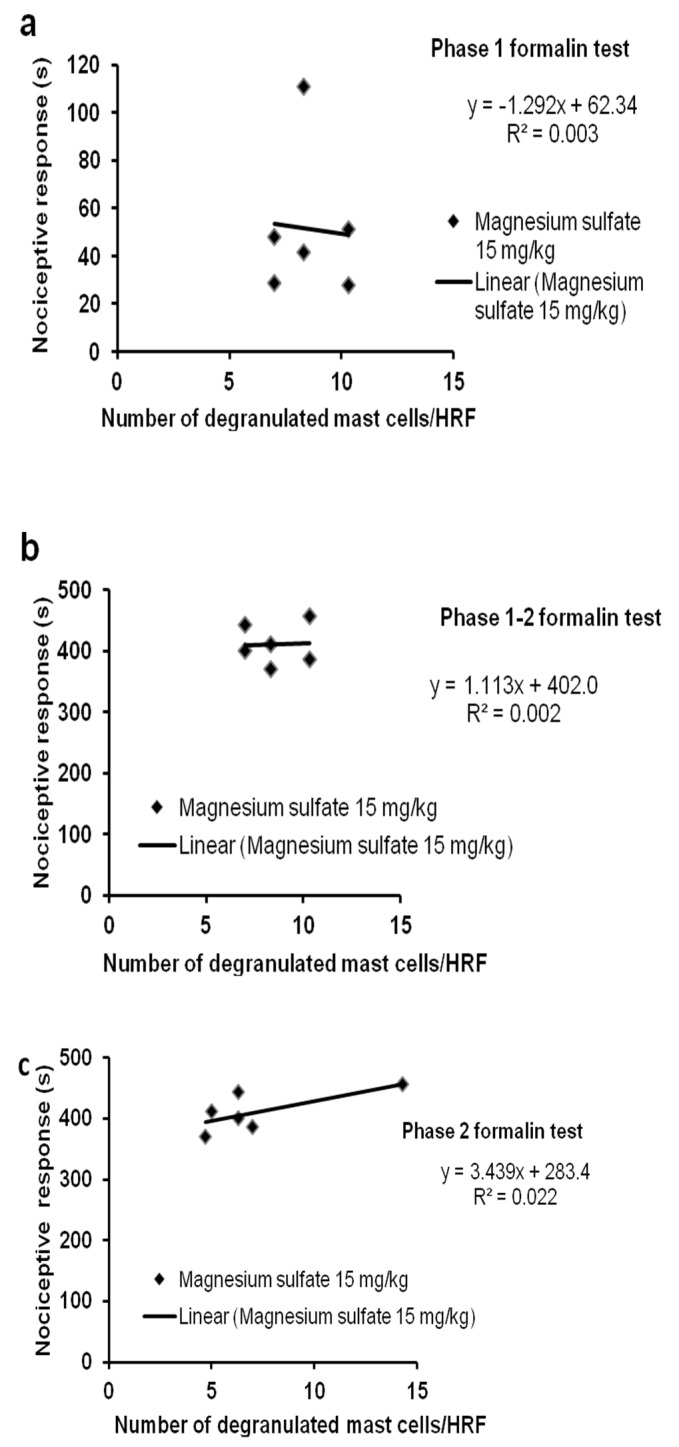
Linear regression analysis of pain and degranulated mast cells after magnesium treatment in the first (**a**), first–second (**b**), and second (**c**) phase of the formalin orofacial test. The nociceptive response (in seconds) was expressed as the total time pain was experienced during the first (**a**) or second (**b**,**c**) phase of the formalin test and compared with the degranulation of mast cells/high representative fields (HRF), during the first (**a**,**b**) or second (**c**) phase of the formalin test. Each point represents the values obtained from one rat (in a total of 6 rats). The calculated Pearson’s correlation coefficients did not show a statistically significant relationship between lip mast cell degranulation and pain after the treatment with magnesium (**a**–**c**).

There were no significant correlations between the formalin-induced degranulation of mast cells and the formalin-induced orofacial pain after treatment with magnesium sulfate (15 mg/kg) (Figure 8). Preventive administration of magnesium sulfate did not affect the correlation between mast cell degranulation at 5 min and pain in the acute phase (r = −0.062, *p* = 0.906) (Figure 8a) or in the second phase (r = 0.050, *p* = 0.925) (Figure 8c) of the formalin test in rats. In addition, there were no significant changes between mast cell degranulation at 25 min and pain in the second phase of the test (r= 0.693, *p* = 0.127) (Figure 8b). Orofacial pain in rats at the 3–6 (r = −0.083, *p* = 0.876) and 24–27 min (r = 0.146, *p* = 0.782; r = 0.399, *p* = 0.434) time intervals was not significantly correlated with degranulation of mast cells at 5 min or at 25 min after injection of formalin and magnesium.

## 3. Discussion

This study, for the first time, shows that: (i) magnesium sulfate and cromoglycate given in combination may prevent the development of inflammatory orofacial pain but not acute nociceptive orofacial pain; (ii) magnesium both enhances or reduces the effect of cromoglycate on pain; (iii) magnesium systemically applied reduced mast cell degranulation in the periphery during the formalin-induced orofacial inflammation (anti-inflammatory effect); (iv) inhibition of degranulation (anti-inflammatory effect) does not significantly contribute to the analgesic effect of magnesium in formalin-induced orofacial pain.

The significance of our work is the increased understanding of the pathophysiological mechanisms of the development of pain and inflammation, the discovery of the target sites of action of new drugs, and the creation of a drug use protocol with the aim of improving the pharmacotherapy of pain and inflammation in the orofacial region.

The formalin-induced orofacial pain is a model of spontaneous pain and is the only animal model with spontaneous persistent cutaneous pain in the trigeminal area [16]. The results of this study suggest that the upper lip (vibrissa pad) of rats is a site rich with mast cells and that injection of different compounds locally reduce the total number of mast cells mechanistically or by stimulated degranulation. The total number of mast cells can change in various diseases because they are inflammatory cells that participate in the body’s defense [32,33,34,35,36]. To elucidate how mast cells degranulate, future research should examine both the protective and destructive effects of a particular disease or the severity of the disease [32,36,37]. In addition, the total number of mast cells may vary depending on drug treatment and/or what is used as a vehicle. In the model of formalin-induced orofacial pain, there is significant degranulation of mast cells in both the acute and inflammatory phases. Results of this study show that the process of degranulation brought by formalin in the upper lip is very rapid and lasts a long time, even when spontaneous pain ceases. In the acute phase of the test, magnesium reduces the number of mast cells, which may explain that osmotically disrupts mast cells by magnesium. We also demonstrated that the physical process of injection of a 100 µL volume of saline to the upper lip per se minimally reduced mast cell number in the first 5 min. It is known that a peripheral painful stimulus leads to the peripheral and central degranulation of mast cells and the occurrence of cranial and viscero pelvic pain [13,37]. In this research, for the first time, we showed the degree of mast cell degranulation on the periphery, locally, at the site of pain and tissue damage, and its connection with the occurrence of pain.

Since we have previously shown that magnesium has a dose-independent analgesic effect [16,23,24], in this study, we used low and high effective doses of magnesium [16]. It is important to note that this effect of magnesium 15 mg/kg is achieved at a dose equal to the daily supplementation dose in humans [25], and those rats did not have hypomagnesemia [16]. Magnesium deficiency can increase the development of pain and inflammation in temporomandibular arthritis pain via an NMDA-dependent mechanism of action [38]. In addition, it is known that low magnesium in the brain is associated with neuroinflammation and neurodegeneration [39]. Recent studies show that low levels of magnesium in rat trigeminal ganglia neurons decreased oxytocin efficacy in reducing headache pain [40]. The analgesic effect of cromoglycate in our study is in accordance with the well-known effect of cromoglycate on the reduction of pain and inflammation [8,13]. Cromoglycate reduces pain in the second phase of the formalin test but does not affect pain in the first phase [8]. Herein, for the first time, we describe its effect on orofacial pain and its possible interaction with magnesium.

The results of this study indicate that magnesium and cromoglycate may interact and that this type of interaction depends on the nature of the pain (acute “pure” nociceptive or inflammatory), as well as the individual effects of both drugs. The addition of magnesium to cromoglycate is not always justified, especially if higher doses of cromoglycate are given. In combination with low-effective doses of cromoglycate, magnesium contributes to its antinociceptive effect in inflammatory pain. In acute “pure” nociceptive pain, it contributes only in a dose for supplementation in humans, while at a lower dose, it antagonizes the effect of cromoglycate. Herein, we show that in the early phase of the formalin test, the combination of cromoglycate and magnesium in a sub-analgesic dose may increase nociception. It is possible that the effect of magnesium dominates. Magnesium at low doses does not reduce “pure” nociceptive pain, and at the same time, activates TRP channels, vanilloid (TRPV1 and TRPV4), and ankyrine (TRPA1) types and releases glutamate and nitric oxide at the periphery [30,41]. All these mechanisms are responsible for the occurrence of “pure” nociceptive pain [42,43,44], and magnesium probably, in this way, enhances the pain in the early phase of the formalin test. Therefore, there may be an interaction between magnesium supplements and other drugs or with combined preparations in which magnesium is used in low doses. However, our results indicate that the administration of magnesium with cromoglicate is not convincingly justified in order to reduce pain because it is not efficient in acute “pure” nociceptive pain, and it reduces inflammatory pain in combination as well as when magnesium is administered itself. Herein, we demonstrated that in orofacial pain, magnesium has dose-independent and low analgesic effects in acute and inflammatory pain and that cromoglycate has a dose-dependent effect with low efficacy in acute “pure” and high efficacy in inflammatory pain. Our results suggest that the systemic use of magnesium or cromoglycate immediately before stomatological intervention or surgery in the orofacial region in the trigeminal area might prevent the onset of pain and inflammation [34,35,36]. According to this, our results may be useful in creating the possible protocol for the use of magnesium as an adjuvant analgesic or anti-inflammatory therapy.

In formalin-induced orofacial pain, the standard opioid, such as morphine, has a dose-dependent and site-specific effect [45]. Morphine reduces pain in the trigeminal region both peripherally and centrally. Intraperitoneally administered morphine dose-dependently reduced pain in the second phase of the orofacial formalin test, while it does not affect pain in phase 1 [46] when administered at the level of the brain stem and hippocampus, morphine reduced pain during both phases of the test [45,47]. Literature data show that magnesium in the formalin pain test exhibits low antinociceptive activity and reduces pain in phase 2 of the formalin test after peripheral and central administration [16,26,48,49,50]. These data suggest that, contrary to morphine, which acts peripherally, spinally, and supraspinally and which can reduce “pure” nociceptive pain, magnesium probably has a primarily peripheral analgesic effect and a weak analgesic effect in “pure” nociceptive pain. Due to this effect, in the formalin test, magnesium has an effect that is more similar to cyclooxygenase inhibitors, reducing pain in phase 2 of the formalin test but not in phase 1 of the formalin test [51,52]. In addition, cromoglycate reduces pain in phase 2 of the formalin test but does not affect pain in phase 1 [8]. Since the second phase of the formalin test reflects central sensitization, this suggests that both magnesium and cromoglycate, when given prophylactically, can prevent central sensitization. Magnesium, as a blocker of the ion channel of NMDA receptors, stops the activation of the NMDA receptor with glutamate [25,48]. Cromoglycate reduced the release of proinflammatory mediators at the periphery and prevented their cumulative actions (of the proinflammatory “soup” of mediators), which led to peripheral and central excitation of neurons. Magnesium has a dose-independent analgesic effect. In phase 2 of the formalin test, magnesium in both tested doses, which were low because they corresponded to doses for supplementation in humans, increased the submaximal analgesic effect of cromoglycate. On the other hand, magnesium reduced the maximum (100%) analgesic effect of cromoglycate probably through the activation of other mechanisms that remained free, e.g., at the periphery by activating TRP receptors, releasing glutamate and nitric oxide, which participated in peripheral and central sensitization [25,48]. Due to the different pharmacological profiles of the drugs, combining analgesics with each other or with adjuvant analgesics may be justified to achieve multimodal analgesia. Although in this research, we showed that magnesium alone at the tested doses has a modest analgesic effect, while the effects of cromoglycate appeared to be more prominent (especially at the highest dose), their combination is not justified from the aspect of analgesia.

This research is significant because it demonstrates the role of magnesium in the neuroinflammatory process and elucidates the anti-inflammatory effect of magnesium. One of the possible mechanisms of the interaction between cromoglycate and magnesium sulfate could be explained by the inhibition of mast cell degranulation and the consequent release of pronociceptive mediators. Cromoglycate is a mast cell stabilizer, while magnesium is an endogenous blocker of calcium channels. Additionally, it modulates the activities of sodium and potassium ion channels and influences membrane potentials [53]. It is well known that mast cells express different ion channels, including Ca^2+^, K^+^, and Cl^−^ channels. Activation of mast cells occurs after the influx of extracellular Ca^2+^ through L-type Ca^2+^ channels, which leads to the release of both preformed mediators and newly synthesized cytokines [54]. The flow of K^+^ and Cl^−^ ions through the cell membrane changes the membrane potential and Ca^2+^ influx [55]. In mast cells, besides voltage-dependent Ca^2+^ channels, store-operated channels (SOCs) contribute to the entry of Ca^2+^ into the cell through Ca^2+^ or other ion SOC channels [56]. Different TRP channels may be involved in Ca^2+^ signaling, such as canonical (TRPC1), melastatin (TRPM), and vanilloid (TRPV) channels in skin mast cells [57,58]. Additionally, TRP channels are non-selective ion channels and can be activated by other cations. The significance of this research is that pathohistological analysis revealed that magnesium sulfate reduces the degranulation of mast cells during tissue damage and inflammation. Our results show that the prophylactic application of magnesium reduces mast cell degranulation by about 20% during the acute phase and about 40% during the inflammatory phase of pain. This anti-degranulatory effect of magnesium confirms that magnesium possesses anti-inflammatory activity. It is well known that magnesium has an anti-inflammatory effect, and possible mechanisms of its actions are decreased production of cytokines (interleukin 6, tumor necrosis factor alpha) [59], NMDA-independent NO modulation, blockade of NMDA receptors [60], etc. Magnesium reduces inflammatory edema as a single drug or in combination with tramadol [27,60]. Anti-edematous effect of magnesium occurs after systemic and local administration [60]. In this study, we showed with a linear regression model that under the action of magnesium, the number of degranulated mast cells in the first and second phases of the formalin test decreased in parallel with the increase in analgesic effect during the second phase of the test, although this correlation was not statistically significant. Our results are supported by the finding that mast cells are not crucial for the development of pain [61,62]. It was shown that mast cell deficiency does not affect pain responses in the paw formalin test [62]. Other researchers have shown that mast cells do not participate in hyperalgesia, which is a consequence of magnesium deficiency [63]. However, we have shown that magnesium reduces mast cell degranulation in vivo, and although this effect does not significantly modify the analgesic effect of magnesium, it probably contributed to its anti-inflammatory effect. Although pain and edema are simultaneous processes in inflammation, some drugs can independently affect pain and edema [27,61], while some drugs, such as cromoglycate, affect both processes simultaneously [64] or separately [63]. In addition, it was shown in inflammatory models that mast cell degranulation is not crucial for the development of mechanical and thermal hyperalgesia, but it is crucial for the development of edema and locally elevated temperature [61]. Taken together, it is possible that separately, both cromoglycate and magnesium have some stabilizing effect on the membranes of neurons and/or mast cells, which mediate independently in analgesia and inflammation. Since magnesium administered as a single drug possesses independent analgesic and anti-degranulatory activities, this may have an important impact on human oral diseases [34,35,36].

By reducing mast cell degranulation in the orofacial region in humans, preventive administration of magnesium in supplementation doses could prevent the onset or progression of periodontal disease [34] and inflammatory diseases of the anterior oral cavity, such as periapical granuloma and periapical cyst gingival hyperplasia [35]. In addition, the use of magnesium could be important during the healing of oral surgical wounds due to the reduction of mast cell degranulation and a consequent anti-inflammatory effect [36]. Our result also suggests that magnesium, by stabilizing mast cell membranes, could reduce morphine-induced mast cell activation and, thus, the onset of paradoxical pain and adverse morphine effects such as pruritus [65]. We can also conclude that magnesium, by reducing mast cell degranulation, non-selectively prevents neuroinflammation. Future immunohistochemical research can confirm the molecular mechanisms involved in pain and inflammation.

This study has some limitations. The nociceptive response for each group was measured only once because the effects were clearly expressed, although, in some situations, they were weak.

## 4. Materials and Methods

### 4.1. Experimental Animals

Male adult rats (Wistar strain, 200–250 g, *n* = 174) were housed in Plexiglas cages (42.5 × 27 × 19 cm; 3 per group). The number of rats per group (*n* = 6) was determined by the intragroup variability in freely available Sample Size & Power Calculator software for the power of 90% and type I error probability of 0.05. Rats were maintained under controlled experimental conditions of temperature (22 ± 1 °C), humidity (60%), and a light/dark cycle (12/12 h). Water and standard rat pellets were freely available. Before use in experiments, rats were acclimatized and habituated to the laboratory and experimental environment. During testing, the animals were unrestrained. During the formalin test, in a separate group of rats, upper lip tissue samples were collected and used for the pathohistological analysis of mast cell number and degranulation. All experiments were performed by the same experimenter at the same time (between 8:00 a.m. and 3:00 p.m.). An experimental rat was used only once. At the end of the experiments, the rats were killed by an intraperitoneal injection of sodium thiopental (200 mg/kg); for pathohistological analysis, they were subsequently decapitated.

### 4.2. Ethics Statement

The experiments were approved by the Institutional Animal Care and Use Committee (Faculty of Medicine University in Belgrade, permission no. 4822/3/2020) and the Republic Ethical Council (Ministry of Agriculture, Forestry and Water Management, approval no. 323-07-11101/2020-05/6 and dated 2020). These ethical institutions operate in accordance with the national Animal Welfare Law and with the European Communities Council Directive 2010/63/EU.

### 4.3. Drugs and Protocol for Drug Administration

Disodium cromoglycate (Cromolyn; Sigma-Aldrich, Milan, Italy) and magnesium sulfate (Magnesio Solfato; S.A.L.F. Spa, Cenate Sotto, BG, Italy) were dissolved in 0.9% NaCl and injected subcutaneously (s.c.) in the back in a total volume of 2 mL/kg. Magnesium sulfate was administered 10 min after the cromoglycate injection. Formalin (1.5% *w*/*v*, 100 μL) was injected s.c. into the right upper lip of rats 5 min after magnesium sulfate. A stock solution of formaldehyde (37%, Gram, Serbia was diluted in 0.9% NaCl to 1.5% formalin (0.55% formaldehyde). The same volume of 0.9% NaCl was administered to the control group to analyze any possible effect of the vehicle.

The effects of prophylactic systemic administration of cromoglycate, magnesium sulfate, or the cromoglycate/magnesium sulfate combination/interaction on pain were investigated in the formalin-induced orofacial pain test. Using the same test, the effect of magnesium sulfate on the total number and degranulation of mast cells in the dermis was examined in a separate group.

### 4.4. The Model of Orofacial Pain

The formalin-induced orofacial pain model was described previously [16]. In brief, formalin (1.5%, 100 μL) was injected into the subcutaneous tissue of the upper lip of the rat, lateral to the nose. The intensity of nociception was registered (in 15 blocks of 3 min) as the total time spent in pain-related behavior (face rubbing/scratching) after the injection of formalin or 0.9% NaCl. This measured the total nociceptive time during the first (0–9 min) and second phases (9–45 min) after formalin injection. Phase 1 represents an acute tonic pain due to peripheral nociceptor sensitization, while phase 2 characterizes an inflammatory pain.

### 4.5. Data Analysis in Pain Testing

The results are expressed as the mean times spent on face rubbing ± standard deviation (SD) obtained in six animals per group (16 groups, total of 96 rats). There were the following groups: cromoglycate (4 groups + 1 vehicle group; *n* = 30), magnesium (2 groups + 1 vehicle group; *n* = 18), cromoglycate + magnesium 5 (3 groups + 1 vehicle group; *n* = 24), and cromoglycate + magnesium 15 (3 groups + 1 vehicle group; *n* = 24). The time course of the effects of individual drugs and their combinations were constructed by plotting the mean time that the animal spent in pain-related behavior as a function of time. In the next step, the antinociceptive effects of drugs were grouped by phases. Treatments that produced decreases in the duration of face rubbing were considered to be antinociceptive.

Antinociceptive activity (AA%) was calculated according to the following formula [16] in each rat:

AA% = ((control rubbing time formalin−post-drug rubbing time):(control rubbing time formalin)) × 100

Percentage inhibition (%I) of the antinociceptive effect of cromoglycate by magnesium sulfate was calculated according to the following formula [24]:

%I = 100 − (% AA with magnesium/% AA without magnesium) × 100

To determine the characteristics of interaction between magnesium sulfate and cromoglycate, a fixed dose analysis and an isobolographic analysis were used [66,67].

Analysis of the interaction between drugs with a low antinociceptive efficacy—Firstly, for a drug with dose-dependent and submaximal effect (up to 60%) (low efficacy drug, cromoglycate in phase 1), the maximal effect was estimated from a double reciprocal plot, and the median effective dose (ED50) value was calculated on this basis [68]. ED50 is a dose that produced a 50% reduction in the number of seconds spent in pain behavior compared with the control group. For a drug with a dose-independent and limited maximal effect (low efficacy drug), a specific dose was chosen (magnesium). The effects of magnesium were examined in a specific dose range, which corresponds to doses for supplementation in humans (based on the literature data [25]). The one low-effective and the one maximum-effective dose tested are chosen for the combination experiments.

Analysis of the interaction between drugs with a high and a low antinociceptive efficacy—Firstly, for a drug with dose-dependent and maximal effect (high efficacy drug, cromoglycate in phase 2), the ED50 value calculated from the corresponding log dose–response curve using the linear regression analysis. For a drug with a dose-independent and limited maximal effect (low efficacy drug), a specific dose was chosen (magnesium). The effects of magnesium were examined in the one low-effective and the one maximum-effective dose.

Afterward, the effects of the magnesium sulfate-cromoglycate combination were examined separately in phases 1 and 2 of the test using ED50 of cromoglycate and its fraction of ED50 with fixed doses of magnesium. To determine the type of interaction, the regression log dose–response curves for cromoglycate alone is compared to the log dose–response curves for cromoglycate in the presence of magnesium sulfate. If there is a significant leftward shift of the log dose–response curves for cromoglycate in the presence of magnesium, the interaction between components is supraadditive (synergistic). If those curves overlap, there is an additive interaction [68]. If there is a significant rightward shift of the log dose–response curves, the interaction between components is antagonistic.

### 4.6. Upper Lip Tissue Collection and Staining

Upper lip tissue samples (*n* = 78) were obtained from 78 rats (6 per group, 13 groups), divided into the naive group (*n* = 6) and into 5 min, 25 min, and 24 h of vehicle (*n* = 18), formalin (*n* = 18), magnesium (*n* = 18) and formalin + magnesium (*n* = 18) groups. The obtained vibrissa tissue (without bones: ~3–5 mm from the injected site) was extracted and fixed in 10% neutral buffered formalin. The tissue was then embedded in paraffin, sectioned at 5 µm thickness, and rehydrated in xylene and then in decreasing concentrations of ethanol. Sections were then stained by the hematoxylin-eosin (HE) method and observed under a light microscope (Olympus optical microscope BH-41, Japan) in order to analyze the morphological preservation and representativeness of tissue samples. After that, the Leishman–Giemsa method was used to identify mast cells, followed by toluidine blue for a detailed analysis of mast cells. Toluidine blue staining (0.1% for 10 min, Sigma-Aldrich, St. Louis, MO, USA) was performed on prepared tissue sections to determine mast cells because it can reliably identify formalin-resistant mast cells present in the dermis [69]. Mast cells were identified by deep blue-purple staining. The researcher that performed the pathohistological analysis was blinded to the labeled experimental groups.

### 4.7. Data Analysis for Mast Cells

Tissue was stained with toluidine blue to avoid confusion of mast cells with other inflammatory cells and because formalin was used as a fixative. Mast cell number and degranulation were counted using image analysis software Image J (version 1.4.3.67). Representative images (digital camera, ArtCam-500MI, Artray, Tokyo, Japan) used for morphological evaluation were obtained at a magnification of 400×. The total number of mast cells and their degranulation (400×; 0.95 mm^2^) were counted on fields randomly chosen as the first three high representative fields (HRF) per one section and then averaged. Mast cells with ≥3 granules outside the cells were considered degranulated. For the purposes of this part of the research, the animals will receive the most effective analgesic dose of magnesium sulfate (15 mg/kg) in this model of pain. The percentage of mast cells with degranulation was calculated and compared to the control. The treatment that caused a statistically significant change in the number of degranulated mast cells was considered significant. The percent inhibition (I%) of mast cell degranulation for each rat by pretreatment was expressed as follows: 

I% = ((number of degranulated control cells − post-treatment number of degranulations)/(number of degranulated controls)) × 100

### 4.8. Statistical Analysis

Behavioral and pathohistological data were expressed as the mean ± SD and were then analyzed by two-way analysis of variance (ANOVA) with repeated measures. Multiple comparisons were performed by Tukey’s HSD post hoc test. In the study of drug interactions, all calculations were performed according to isobologram formulas [67,68,69]. For the comparison of two regression lines, the test for parallelism and the relative potency test was used [69]. When 95% of the confidence limits (CL) did not overlap 1.0, it was considered that the potency ratio was statistically significant (*p* < 0.05). Correlation analysis between mast cell degranulation and pain under the effect of magnesium sulfate was performed with Pearson’s correlation coefficient (r). A *p* < 0.05 was considered statistically significant.

## 5. Conclusions

Our results indicate that the systemic and prophylactic administration of cromoglycate alone can be an effective adjuvant analgesic therapy in the trigeminal region. The effect of a combination/interaction of cromoglycate and magnesium sulfate depends on the nature of the pain (“pure” nociceptive or inflammatory), as well as the individual effects of both drugs. A combination of magnesium–cromoglycate is not of great therapeutic importance in the treatment of pain because the analgesic effect is canceled or increased to the extent that can be achieved by their separate application. Magnesium reduced the degranulation of mast cells in the trigeminal area in the model of orofacial pain, which contributes to its anti-inflammatory effect. The inhibition of degranulation occurred in both the early acute and the inflammatory stages and was not observed during the later time of injury. The analgesic effect of magnesium was associated with the degranulation of mast cells (anti-inflammatory effect), but there was no significant correlation between these two processes.

## Data Availability

The data presented in this study are available on request from the corresponding author. The data are not publicly available due to privacy reason.
